# Factors of the childbirth fear among nulliparous women in Iran

**DOI:** 10.1186/s12884-022-04870-1

**Published:** 2022-07-06

**Authors:** Safieh Kananikandeh, Farkhondeh Amin Shokravi, Mojgan Mirghafourvand, Shayesteh Jahanfar

**Affiliations:** 1 Health Education and Health Promotion, Department of Health Education and Health Promotion, Faculty of Medical Sciences, Tarbiat Modares University, Tehran, Iran; 2 Reproductive Health, Social Determinants of Health Research Center, Faculty of Nursing and Midwifery, Tabriz University of Medical Sciences, Tabriz, Iran; 3 Department of Public Health and Community Medicine, Tufts University School of Medicine, Boston, USA

**Keywords:** Fear of childbirth, Pregnancy, Nulliparous

## Abstract

**Background:**

Fear of childbirth is an anxiety associated with childbirth, which manifests itself in physical and concentration problems. It is often associated with requesting a cesarean section, and it is prevalent in nulliparous women. This is a study aimed to summarize the published research on the factors for fear of childbirth in nulliparous women in Iran.

**Methods:**

This study was conducted based on the PRISMA statement. A literature search was performed on nine electronic databases (Web of Sciences, Since Direct, Scopus, PubMed, Cochrane Library, ProQuest, and Persian databases including Scientific Information Database, Irandoc, and Magiran) using keywords related to fear of childbirth, factors, nulliparous, and Iran from 2000 to 2020. This study included cross-sectional studies with full-text in English or Persian in Iran. The quality of the selected studies was evaluated independently by two authors and via the STROBE checklist.

**Results:**

In this study, 93 articles were identified,13 duplicate articles were excluded, 80 articles were screened by title and abstract, 62 were excluded, and the full-text of 18 articles was assessed for analysis. Of these, 12 were excluded, and six articles were reviewed. Six studies were conducted in different provinces of Iran. Based on the study results, factors of the fear of childbirth in nulliparous women were: biological (the process of labor and childbirth and labor pain, concern for the baby (harm to the baby and baby infirmity), psychological (painful injections during labor and suturing in childbirth), and individual (loss of control during labor).

**Conclusions:**

This study identified four main factors that affect fear of childbirth status in nulliparous women, and concern for the baby was a more common factor in this study. In conclusion, these factors can be reduced by increasing their assurance about child health, training during pregnancy, talking about positive experiences, and holding workshops.

## Background

Childbirth is a natural process. It is considered a critical event in the life of a woman. This unpredictable process may be associated with complications and risk of death for both mother and child. Moreover, it affects a woman's thoughts and feelings. These feelings involve happiness and confidence to concern and fear [1–8].

Fear of childbirth (FOC) is an anxiety associated with expected childbirth. FOC manifests itself in phobias, nightmares, physical problems, and concentration problems. It is often related to requesting a cesarean section (CS) [9–13].

In recent years, FOC has increased. According to previous reports, FOC has been found in about 20% of pregnant women. Globally, the prevalence of FOC has varied from 5% to more than 40%. According to existing studies, this prevalence has been reported in European countries from 6.3 to 14.8%. It is also estimated that 25% of pregnant women have FOC in Iran [4, 5, 13–16].

In general, FOC may have several risks, such as abortion, posttraumatic stress disorder, depression, risk of birth trauma such as fistula, risk of dystocia, hypertension and preeclampsia, preterm labor, labor intolerable pain, reduced quality of life, as well as harmful effects on the children such as low birth weight and impaired immune system. Therefore, FOC and its complications are likely to increase obstetric interventions and subsequent health care costs [9, 13, 15–21].

Various studies have identified various potential causes of FOC, including the following: personality traits, concerns about baby health, low educational status, history of childhood sexual and physical violence, inadequate awareness, interactions with medical staff, fear of the unknown, birth-related problems and procedures, internalizing other women's negative stories, being a nulliparous, rural living, low self-efficacy, lack of social support, cultural differences, race and ethnicity, and self-esteem [1, 4, 8, 13, 22–24].

Therefore, it is noteworthy that FOC affects the women's decision to choose the delivery method and is more prevalent in nulliparous women. Findings of a cohort study by Räisänen et al. (2014) revealed that CS rates for women with FOC were more than four times higher than those without FOC. Based on the available meta-analysis study conducted in Iran, FOC was the first cause of CS. Also, FOC has a multidimensional nature and varies in different regions with different socio-cultural characteristics, and assessment of individual needs to reduce FOC should be considered [4, 13, 23, 25, 26].

According to the literature, it seems necessary to conduct a study to identify the factors of FOC among nulliparous women in Iranian culture. Moreover, a review was a requirement to proceed with the next stage of this study, a future clinical trial in this area in Iran. Hence, this study aimed to investigate FOC factors among nulliparous women in Iran. Hopefully, the results of this study will be helpful as a phase from the main research of the authors in designing educational content for a future clinical trial to manage and reduce FOC and consequently reduce the rate of CS.

## Methods

This study was conducted based on the PRISMA statement. The study's research question was the factors related to the FOC among Iranian nulliparous pregnant women are.

### Search strategy

A literature search was performed on electronic databases, including Web of Sciences (WOS), Since Direct, Scopus, PubMed, Cochrane Library, ProQuest, and Persian databases (Scientific Information Database (SID), Irandoc, and Magiran) from January 2000 to December 2020 due to the irrelevance of studies in this field before this period without time limitation based on the literature review. Restrictions applied to the collection of articles and studies searched. So that only English and Persian articles and primary studies were selected.

These keywords were used in the search strategy: "factors" OR "risk factors" OR "causes" OR "reasons" OR "determinants" AND "fear of childbirth" OR "childbirth fear" OR "childbirth-related fear" OR "fear of birth" OR "birth fear" OR "fear of parturition" OR "parturition fear" OR "fear of delivery" OR "delivery fear" OR "fear of labor" OR "labor fear" AND "nulliparous" OR "primiparous" OR "primigravidae" OR "primipara" AND "Iran". Finally, the bibliographies of related articles were manually searched for possible studies that could have been omitted.

Tokophobia was not included in the search terms because it as FOC sometimes occurs before pregnancy and the fear begins in adolescence or early adulthood (primary tokophobia) and sometimes occurs after having a negative or traumatic birth experience (secondary tokophobia), but FOC in this study referred to FOC during pregnancy among nulliparous pregnant women [7, 21].

### Inclusion criteria

#### Types of participants

Participants were nulliparous pregnant women.

#### Types of studies

The studies included cross-sectional studies with full-text available in English or Persian in Iran.

#### Types of outcomes

The outcome was factors of FOC, and publications examining the fear of postpartum were excluded.

### Selection of studies

**Fig. 1:**
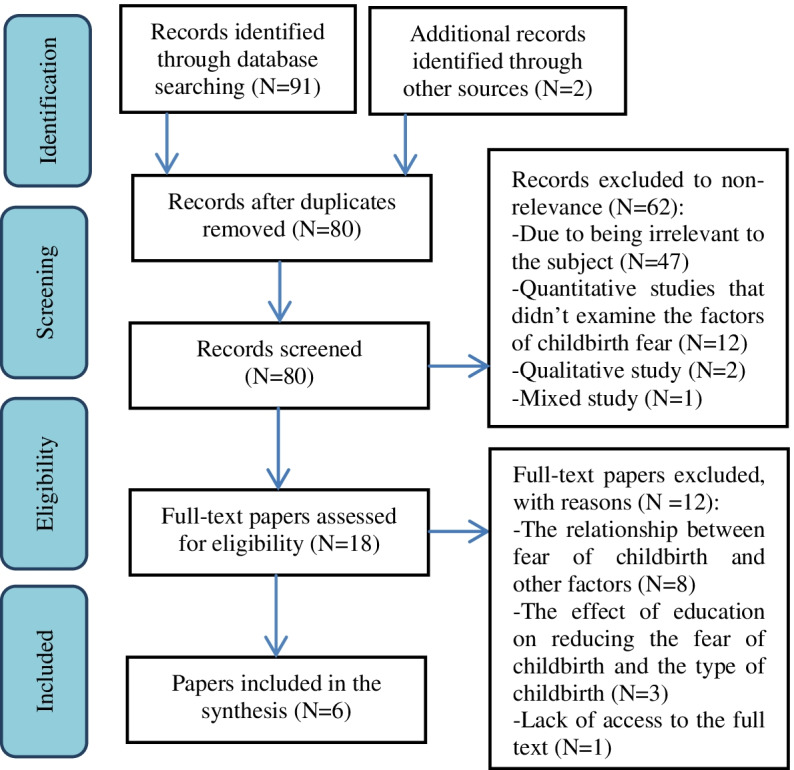
PRISMA flow diagram

Importing articles and deleting duplicates was accomplished using the Mendeley program. The title and abstracts were screened for relevance by two independent reviewers. Full-text articles were screened further for eligibility and were evaluated for quality by two reviewers. Disagreements and irrelevant articles were examined and resolved by consensus; inappropriate and irrelevant articles were excluded. Figure 1 shows the process of selecting articles based on the 2020 PRISMA diagram [27].

## Results

### Search outcome

At first, using the search strategy, 93 articles were found, 13 duplicate articles were excluded. Then 80 articles were screened by title and abstract after removing duplicates, and 62 of them were excluded. Of the remaining 18 articles, full-text articles were read, and 12 of these were subsequently excluded. Finally, six articles related to the topic of factors of FOC in nulliparous women in Iran with a sample size of 1711 participants were reviewed (Fig. 1). The STROBE checklist was also approved for the six cross-sectional studies [28].

### Description of studies

This study showed that studies had been conducted in different provinces of Iran, including the northern, central, western, southeastern, and northeastern provinces. Three studies were carried out on FOC causes, one paper on the fears related to pregnancy and childbirth, and two articles on the relationship between FOC and delivery mode. Different questionnaires were used in these studies (Table 1).

**Table 1:** Characteristics of included studies

Author, year	Study purpose	Type of study	Sample size	Tool	County	Results	STROBE checklist score
Taheri et al. 2015 [29]	To investigate the causes of FOC in pregnant women in Shahrekord	Cross-sectional	130	FOC Questionnaire	Shahrekord	The main causes of FOC in pregnant women were fear of painful injections during labor, suturing in childbirth, labor pain, and loss of control during labor	22
Khorsandi et al. (2014) [30]	To investigate the causes and factors related to natural childbirth among pregnant women who attended prenatal care of Arak	Cross-sectional	595	Lowe Questionnaire	Arak	The most common causes of fear were possible harm to the baby, baby infirmity, and labor pain, respectively	30
Farajzad touli et al. (2018) [31]	To assess prevalence and causes of FOC in primiparous pregnant women	Cross-sectional	181	Harman Childbirth Attitude Questionnaire	Talesh	The most common cause of FOC was harm to the baby. Fear of baby infirmity and fear of labor was in the second and third ranks	34
Matinnia et al. (2015) [32]	To examine the content of maternal fear and the associated demographic factors in a sample of Iranian primigravidae	Cross-sectional	342	FOC Questionnaire	Hamedan	The most frequent factor for all the respondents was 'fear related to the process of labor and childbirth' These factors were included labor pain, vaginal rupturing, being injured during childbirth, losing one's own life during childbirth, an unpredictable problem during childbirth, prolonged labor, and delivery, and ending up with emergency interventions	22
Andaroon et al. (2017) [33]	To determine the relationship between the intensity of FOC and choosing the type of childbirth in primiparous women	Cross-sectional	220	Wijma FOC Questionnaire	Mashhad	The most common cause of FOC was fear of labor pain	28
Negahban et al. (2009) [34]	To assess the relationship between fears from vaginal delivery with the occurrence of emergency cesarean in primiparous women	Cross-sectional	243	Structured Questionnaire	Rafsanjan	The most mentioned reasons for FOC were labor pain and harm to the baby, respectively	24

Based on the study results, determinants of the FOC in nulliparous women were: biological factor (the process of labor and childbirth and labor pain), concern for the baby factor (harm to the baby and baby infirmity), psychological factor (painful injections during labor and suturing in childbirth), and individual factor (loss of control during labor) [29–34].

Taheri et al. (2015) research was conducted on the causes of FOC among pregnant women in Shahrekord. The results showed that 94.61% of mothers stated that they were afraid of painful injections during childbirth, 91.53% of suturing in childbirth, 87.71% of labor pain, and 62.31% due to loss of control during labor. Based on the findings, the mean FOC score was significantly higher in the nulliparous women than in multiparous women. Nightmare, harm to the baby, baby disability, hospital environment, bleeding, being left alone, not receiving care, not cooperating with the doctor, losing control, delivery anxiety, and labor pain, respectively, were other factors [29].

A cross-sectional study by Khorsandi et al. (2014) was conducted to investigate different factors of fear in vaginal delivery among pregnant women in Arak. The mean score for nulliparous women on the FOC questionnaire was significantly higher than in multiparous women. There was a significant difference between the birth scores of women with vaginal childbirth and women with cesarean section. Furthermore, the results indicated that the most common causes of FOC were the possible harm to the baby, baby infirmity, and labor pain, respectively. Other reasons were included assessing delivery anxiety, giving birth, losing control, suturing, being alone, lack of cooperation with the physician, not receiving care, bleeding, hospital environment, injections, and nightmare [30].

A study by Farajzad touli et al. on prevalence and causes of FOC in nulliparous women showed that 82.7% of mothers had a degree of FOC. There was a significant difference between the knowledge of the complications of cesarean section and the mean score of FOC, as FOC reduced with increased awareness. In addition, the most common cause of FOC was harm to the baby; fear of baby infirmity and fear of labor was in the second and third ranks. Other reasons included the following, respectively: labor anxiety, losing control, being alone, suturing, not receiving care, lack of cooperation with midwife or physician, injections, labor pain, bleeding, hospital environment, and nightmare [31].

The study of Matinnia et al. (2015) was conducted to investigate the content of maternal fear and the associated demographic factors in a sample of Iranian nulliparous. According to the study results, all nulliparous reported some degree of fear. Six categories were identified. The most frequent common factor for all the respondents was ‛fear related to the process of labor and childbirth, followed by health and life of the baby, competence and behavior of maternity ward personnel, own capabilities and reactions, becoming a parent and family life after deliver, and general fear in pregnancy’ [32].

The study of Andaroon et al. (2017) was performed to determine the relationship between FOC with choosing the mode of delivery in primiparous women referred to the health centers of Mashhad. According to the study results, there was a significant association between selecting the mode of delivery and the intensity of FOC. The majority of women with intense FOC had selected cesarean section. Moreover, the most common cause of FOC was fear of labor pain, followed by baby health, hearing the experiences of others, physical complications, and lack of trust in employees, respectively [33].

Negahban et al. (2009) studied the relationship between fear from vaginal delivery with the occurrence of emergency cesarean in primiparous women. The results showed a high percentage of women expressed an intense fear of natural childbirth. There was a significant relationship between fear and the method of delivery. Moreover, the most mentioned reasons for FOC were labor pain and harm to the baby, respectively. The other causes of FOC were the suggestion of those around the person, vaginal examination, maternal injury, and the cry of other women [34].

## Discussion

This study was conducted to investigate the factors associated with FOC in nulliparous women in Iran. Six articles on the factors of FOC in nulliparous women were reviewed for this study. The overall results of this study showed factors of the FOC in nulliparous women were biological factors including the process of labor and childbirth and labor pain, concern for the baby factor including harm to the baby and baby infirmity, psychological factors including painful injections during labor and suturing in childbirth, and individual factors including loss of control during labor.

According to the present study's findings, one of the factors influencing the FOC in nulliparous women was the biological factor with sub-factors the process of labor and childbirth and labor pain. So that fear of pain and the process of labor and childbirth in half of the reviewed studies was the first common cause of FOC, and in the other half was the third [29–34]. These findings were consistent with the results of other studies; the fear of pain was the first, the second, and the third cause of FOC in these studies, respectively [35–37]. Similarly, Serçekuş et al. argued that the most common reason for FOC in primiparous women is fear of labor pain [38]. In comparison, in the study of Tsui et al., the first factor identified for the cause of FOC was negative stories followed by negative attitudes or moods. In her study, a quarter of the study population was multiparous women [39]. However, studies consistent with our study have been performed among nulliparous women, except for 10% of the study sample of Demšar et al. [35–37]. Therefore, the study's target population has probably been influential in the common cause of FOC. Moreover, although in the study of Mortazavi et al., FOC did not differ in terms of parity, nonetheless, women's experiences of FOC are influenced by many individuals and social patterns, including negative stories, negative attitudes, mood-related aspects, and a previous negative experience of childbirth as the main causes of FOC in multiparous women and fear of pain as one of the common causes of FOC in nulliparous women likely because of the first experience [24, 25, 39–41].

Concern for the baby factor with sub-factors of the harm to the baby and baby infirmity was another factor of FOC among nulliparous women in this study. These were common reasons for FOC in the studies conducted by Khorsandi et al., Farajzad touli et al., and Negahban et al. [30, 31, 34]. This finding was congruent with a study by Saisto et al. and Abd El-Aziz et al. [35, 36]. By contrast, harm to the baby and baby infirmity were not found as a common cause of FOC in other studies in nulliparous women [25, 37–39, 41–45]. In this study, concern for the baby factor as a common cause of FOC can be interpreted as follows:

1) The study's participants were nulliparous women without experience of childbirth. They most likely worried about their baby's health after hearing terrible stories about the negative impact of birth on the baby's health. As the study finding by Sen et al. showed the 69% of women who had heard about their relatives having a bad birth were affected by these experiences [46], 2) Knowing a woman who has delivered an injured or disabled child, and 3) This child was a golden baby for them because he/she was their firstborn. Hence, they are more concerned about his/her health. To that end, in a study by Ternström et al., the expectation of the birth of the first child was reported as a factor associated with FOC among pregnant women [47].

In line with the results of this study, psychological factors (including painful injections during labor and suturing in childbirth) were one of the factors of FOC in nulliparous women, no study was found [29]. Only in a study, fear of needles was one of FOC causes, which was a less common finding [37]. It can be argued that the psychological factor may include different sub-factors in other studies, or the psychological factor may be a sub-factor of another factor [43]. Regarding this finding, it seems that nulliparous women are afraid of the intervention of medical staff in the labor process in Iran. This fear will probably be managed if more attention is paid to the relationship between the medical staff and the patient in medical settings.

In addition to the above, the present study showed the individual factor (loss of control during labor) was the last factor of the FOC among nulliparous women [29]. In agreement with this finding, Lowe found fear of losing control during delivery was most frequently cited by primiparous women [48]. Furthermore, in the research by Geissbuehler et al., fear of losing control was one of the most frequent answers concerning FOC, which confirms the present study finding [49]. Nonetheless, in a study by Demšar et al., ‛fear of having no control over the situation’ and ‛fear of losing control during birth’ were the second and eighth most common factors of FOC [37]. Therefore, it is noteworthy that in this study, nulliparous women may have this fear due to lack of experience of vaginal childbirth, unfamiliarity with the labor process, and lack of skills to manage the situation. With this in mind, it is possible to strengthen the individual skills of pregnant women in this field by holding problem-solving workshops, talking about positive experiences, and having group discussions.

In general, studies showed that FOC reasons are divided into several categories: social factors (e.g., lack of social support), biological factors (e.g., fear of pain), psychological factors (e.g., mental health problems, past traumatic events, and fear of being a parent) or secondary factors (e.g., previous childbirth experiences) [5, 11, 22, 50]. Some of these factors were also evident in our study. But factors related to fear of harm to the baby and inability during labor were more common in the current study. Based on studies, women's experiences of FOC are also influenced by cultural contexts and environments [4, 46, 50]. Therefore, it seems that this fear can be overcome by creating the right insight [51].

### Limitation

FOC study was limited to nulliparous women in Iran, which was a requirement to proceed with the next stage of this study, a future clinical trial in this area in Iran. Although we tried to include all eligible studies based on our review objective, there is a possibility some studies were lost unintentionally.

## Conclusion

This study focuses on the factors of FOC in nulliparous women in Iran. Based on the study results of the factors of the FOC in nulliparous women were biological (the process of labor and childbirth and labor pain), concern for the baby (harm to the baby and baby infirmity), psychological (painful injections during labor and suturing in childbirth), and individual (loss of control during labor). It was observed concern for the baby factor was more common in this study. In conclusion, the women's FOC regarding these factors can be reduced by increasing their assurance about child health, providing appropriate training during pregnancy, explaining the whole process of childbirth and making it more accessible, raising maternal awareness about pain-relief methods during labor in hospitals, talking about positive experiences, holding problem-solving and enabling workshops, improving the interaction between medical staff and pregnant women, as well as providing appropriate conditions for further care and support after birth. In future research, the authors intend to concentrate on conducting a qualitative study in this area to complement the findings of this study.

## Data Availability

All data used and analyzed during this study are included in this published article (Table 1).

## Appendix

### Search strategy

For each database, keywords and combinations were further updated to improve search results. To that end, the search formula for the PubMed database was: ((((((((((((((((((((factors [Title/Abstract]) OR (risk factors [Title/Abstract])) OR (causes[Title/Abstract])) OR (reasons[Title/Abstract])) OR (determinants[Title/Abstract])) AND (fear of childbirth[Title/Abstract])) OR (childbirth fear[Title/Abstract])) OR (childbirth-related fear[Title/Abstract])) OR (fear of birth[Title/Abstract])) OR (birth fear[Title/Abstract])) OR (fear of parturition[Title/Abstract])) OR (parturition fear[Title/Abstract])) OR (fear of delivery[Title/Abstract])) OR (delivery fear[Title/Abstract])) OR (fear of labor[Title/Abstract])) OR (labor fear[Title/Abstract])) AND (nulliparous[Title/Abstract])) OR (primiparous[Title/Abstract])) OR (primigravidae[Title/Abstract])) OR (primipara[Title/Abstract])) AND (Iran[Title/Abstract])))))))))))))))))))).

## Abbreviations

FOC: Fear of childbirth
CS: Cesarean section
